# Shared genetic basis between genetic generalized epilepsy and background electroencephalographic oscillations

**DOI:** 10.1111/epi.16922

**Published:** 2021-05-18

**Authors:** Remi Stevelink, Jurjen J. Luykx, Bochao D. Lin, Costin Leu, Dennis Lal, Alexander W. Smith, Dick Schijven, Johannes A. Carpay, Koen Rademaker, Roiza A. Rodrigues Baldez, Orrin Devinsky, Kees P. J. Braun, Floor E. Jansen, Dirk J. A. Smit, Bobby P. C. Koeleman, International League Against Epilepsy Consortium on Complex Epilepsies, Bassel Abou‐Khalil, Pauls Auce, Andreja Avbersek, Melanie Bahlo, David J. Balding, Thomas Bast, Larry Baum, Albert J. Becker, Felicitas Becker, Bianca Berghuis, Samuel F. Berkovic, Katja E. Boysen, Jonathan P. Bradfield, Lawrence C. Brody, Russell J. Buono, Ellen Campbell, Gregory D. Cascino, Claudia B. Catarino, Gianpiero L. Cavalleri, Stacey S. Cherny, Krishna Chinthapalli, Alison J. Coffey, Alastair Compston, Antonietta Coppola, Patrick Cossette, John J. Craig, Gerrit‐Jan de Haan, Peter De Jonghe, Carolien G. F. de Kovel, Norman Delanty, Chantal Depondt, Dennis J. Dlugos, Colin P. Doherty, Christian E. Elger, Johan G. Eriksson, Thomas N. Ferraro, Martha Feucht, Ben Francis, Andre Franke, Jacqueline A. French, Saskia Freytag, Verena Gaus, Eric B. Geller, Christian Gieger, Tracy Glauser, Simon Glynn, David B. Goldstein, Hongsheng Gui, Youling Guo, Kevin F. Haas, Hakon Hakonarson, Kerstin Hallmann, Sheryl Haut, Erin L. Heinzen, Ingo Helbig, Christian Hengsbach, Helle Hjalgrim, Michele Iacomino, Andrés Ingason, Jennifer Jamnadas‐Khoda, Michael R. Johnson, Reetta Kälviäinen, Anne‐Mari Kantanen, Dalia Kasperavičiūte, Dorothee Kasteleijn‐Nolst Trenite, Heidi E. Kirsch, Robert C. Knowlton, Roland Krause, Martin Krenn, Wolfram S. Kunz, Ruben Kuzniecky, Patrick Kwan, Yu‐Lung Lau, Anna‐Elina Lehesjoki, Holger Lerche, Wolfgang Lieb, Dick Lindhout, Warren D. Lo, Iscia Lopes‐Cendes, Daniel H. Lowenstein, Alberto Malovini, Anthony G. Marson, Thomas Mayer, Mark McCormack, James L. Mills, Nasir Mirza, Martina Moerzinger, Rikke S. Møller, Anne M. Molloy, Hiltrud Muhle, Mark Newton, Ping‐Wing Ng, Markus M. Nöthen, Peter Nürnberg, Terence J. O’Brien, Karen L. Oliver, Aarno Palotie, Faith Pangilinan, Sarah Peter, Slavé Petrovski, Annapurna Poduri, Michael Privitera, Rodney Radtke, Sarah Rau, Philipp S. Reif, Eva M. Reinthaler, Felix Rosenow, Josemir W. Sander, Thomas Sander, Theresa Scattergood, Steven C. Schachter, Christoph J. Schankin, Ingrid E. Scheffer, Bettina Schmitz, Susanne Schoch, Pak C. Sham, Jerry J. Shih, Graeme J. Sills, Sanjay M. Sisodiya, Lisa Slattery, David F. Smith, Michael C. Smith, Philip E. Smith, Anja C. M. Sonsma, Doug Speed, Michael R. Sperling, Bernhard J. Steinhoff, Ulrich Stephani, Konstantin Strauch, Pasquale Striano, Hans Stroink, Rainer Surges, K. Meng Tan, Liu Lin Thio, G. Neil Thomas, Marian Todaro, Rossana Tozzi, Maria S. Vari, Eileen P. G. Vining, Frank Visscher, Sarah von Spiczak, Nicole M. Walley, Yvonne G. Weber, Zhi Wei, Ruta Mameniskiene, Judith Weisenberg, Christopher D. Whelan, Peter Widdess‐Walsh, Markus Wolff, Stefan Wolking, Wanling Yang, Federico Zara, Fritz Zimprich, Epi25 Collaborative, Yen‐Chen Anne Feng, Daniel P. Howrigan, Liam E. Abbott, Katherine Tashman, Felecia Cerrato, Dennis Lal, Claire Churchhouse, Namrata Gupta, Stacey B. Gabriel, Mark J. Daly, Eric S. Lander, Benjamin M. Neale, Samuel F. Berkovic, Holger Lerche, David B. Goldstein, Daniel H. Lowenstein, Gianpiero L. Cavalleri, Patrick Cossette, Chris Cotsapas, Peter De Jonghe, Tracy Dixon‐Salazar, Renzo Guerrini, Hakon Hakonarson, Erin L. Heinzen, Ingo Helbig, Patrick Kwan, Slavé Petrovski, Sitharthan Kamalakaran, Sanjay M. Sisodiya, Randy Stewart, Sarah Weckhuysen, Dennis J. Dlugos, Ingrid E. Scheffer, Pasquale Striano, Catharine Freyer, Roland Krause, Patrick May, Kevin McKenna, Brigid M. Regan, Susannah T. Bellows, Caitlin A. Bennett, Esther M.C. Johns, Alexandra Macdonald, Hannah Shilling, Rosemary Burgess, Dorien Weckhuysen, Melanie Bahlo, Terence J. O'Brien, Marian Todaro, Hannah Stamberger, Chantal Depondt, Danielle M. Andrade, Tara R. Sadoway, Kelly Mo, Heinz Krestel, Sabina Gallati, Savvas S. Papacostas, Ioanna Kousiappa, George A. Tanteles, Katalin Štěrbová, Markéta Vlčková, Lucie Sedláčková, Petra Laššuthová, Karl Martin Klein, Felix Rosenow, Philipp S. Reif, Susanne Knake, Wolfram S. Kunz, Gábor Zsurka, Christian E. Elger, Jürgen Bauer, Michael Rademacher, Manuela Pendziwiat, Hiltrud Muhle, Annika Rademacher, Andreas van Baalen, Sarah von Spiczak, Ulrich Stephani, Zaid Afawi, Amos D. Korczyn, Moien Kanaan, Christina Canavati, Gerhard Kurlemann, Karen Müller‐Schlüter, Gerhard Kluger, Martin Häusler, Ilan Blatt, Johannes R. Lemke, Ilona Krey, Yvonne G. Weber, Stefan Wolking, Felicitas Becker, Christian Hengsbach, Sarah Rau, Ana F. Maisch, Bernhard J. Steinhoff, Andreas Schulze‐Bonhage, Susanne Schubert‐Bast, Herbert Schreiber, Ingo Borggräfe, Christoph J. Schankin, Thomas Mayer, Rudolf Korinthenberg, Knut Brockmann, Dieter Dennig, Rene Madeleyn, Reetta Kälviäinen, Pia Auvinen, Anni Saarela, Tarja Linnankivi, Anna‐Elina Lehesjoki, Mark I. Rees, Seo‐Kyung Chung, William O. Pickrell, Robert Powell, Natascha Schneider, Simona Balestrini, Sara Zagaglia, Vera Braatz, Anthony G. Marson, Michael R. Johnson, Pauls Auce, Graeme J. Sills, Larry W. Baum, Pak C. Sham, Stacey S. Cherny, Colin H.T. Lui, Nina Barišić, Norman Delanty, Colin P. Doherty, Arif Shukralla, Mark McCormack, Hany El‐Naggar, Laura Canafoglia, Silvana Franceschetti, Barbara Castellotti, Tiziana Granata, Federico Zara, Michele Iacomino, Francesca Madia, Maria Stella Vari, Maria Margherita Mancardi, Vincenzo Salpietro, Francesca Bisulli, Paolo Tinuper, Laura Licchetta, Tommaso Pippucci, Carlotta Stipa, Raffaella Minardi, Antonio Gambardella, Angelo Labate, Grazia Annesi, Lorella Manna, Monica Gagliardi, Elena Parrini, Davide Mei, Annalisa Vetro, Claudia Bianchini, Martino Montomoli, Viola Doccini, Carla Marini, Toshimitsu Suzuki, Yushi Inoue, Kazuhiro Yamakawa, Birute Tumiene, Lynette G. Sadleir, Chontelle King, Emily Mountier, S. Hande Caglayan, Mutluay Arslan, Zuhal Yapıcı, Uluc Yis, Pınar Topaloglu, Bulent Kara, Dilsad Turkdogan, Aslı Gundogdu‐Eken, Nerses Bebek, Sibel Uğur‐İşeri, Betül Baykan, Barış Salman, Garen Haryanyan, Emrah Yücesan, Yeşim Kesim, Çiğdem Özkara, Annapurna Poduri, Russell J. Buono, Thomas N. Ferraro, Michael R. Sperling, Warren Lo, Michael Privitera, Jacqueline A. French, Steven Schachter, Ruben I. Kuzniecky, Manu Hegde, Pouya Khankhanian, Katherine L. Helbig, Colin A. Ellis

**Affiliations:** ^1^ Department of Genetics UMC Utrecht Brain Center University Medical Center Utrecht Utrecht University Utrecht the Netherlands; ^2^ Department of Neurology UMC Utrecht Brain Center University Medical Center Utrecht Utrecht University Utrecht the Netherlands; ^3^ Department of Psychiatry UMC Utrecht Brain Center University Medical Center Utrecht Utrecht University Utrecht the Netherlands; ^4^ Department of Translational Neuroscience UMC Utrecht Brain Center University Medical Center Utrecht Utrecht University Utrecht the Netherlands; ^5^ GGNet Mental Health Apeldoorn the Netherlands; ^6^ Broad Institute of Harvard and Massachusetts Institute of Technology Cambridge Massachussets USA; ^7^ Department of Neurology Tergooi Hospital Hilversum the Netherlands; ^8^ Clinical Research Laboratory on Neuroinfectious Diseases Evandro Chagas Clinical Research Institute Oswaldo Cruz Foundation Rio de Janeiro Brazil; ^9^ Comprehensive Epilepsy Center New York University School of Medicine New York New York USA; ^10^ Psychiatry Department Amsterdam Neuroscience Amsterdam Medical Center, University of Amsterdam Amsterdam the Netherlands

**Keywords:** beta power, EEG, generalized epilepsy, GGE, oscillations, PRS

## Abstract

**Objective:**

Paroxysmal epileptiform abnormalities on electroencephalography (EEG) are the hallmark of epilepsies, but it is uncertain to what extent epilepsy and background EEG oscillations share neurobiological underpinnings. Here, we aimed to assess the genetic correlation between epilepsy and background EEG oscillations.

**Methods:**

Confounding factors, including the heterogeneous etiology of epilepsies and medication effects, hamper studies on background brain activity in people with epilepsy. To overcome this limitation, we compared genetic data from a genome‐wide association study (GWAS) on epilepsy (*n* = 12 803 people with epilepsy and 24 218 controls) with that from a GWAS on background EEG (*n* = 8425 subjects without epilepsy), in which background EEG oscillation power was quantified in four different frequency bands: alpha, beta, delta, and theta. We replicated our findings in an independent epilepsy replication dataset (*n* = 4851 people with epilepsy and 20 428 controls). To assess the genetic overlap between these phenotypes, we performed genetic correlation analyses using linkage disequilibrium score regression, polygenic risk scores, and Mendelian randomization analyses.

**Results:**

Our analyses show strong genetic correlations of genetic generalized epilepsy (GGE) with background EEG oscillations, primarily in the beta frequency band. Furthermore, we show that subjects with higher beta and theta polygenic risk scores have a significantly higher risk of having generalized epilepsy. Mendelian randomization analyses suggest a causal effect of GGE genetic liability on beta oscillations.

**Significance:**

Our results point to shared biological mechanisms underlying background EEG oscillations and the susceptibility for GGE, opening avenues to investigate the clinical utility of background EEG oscillations in the diagnostic workup of epilepsy.

**Key Points**

- Genetic correlation studies show shared genetic underpinnings between GGE and power of background oscillations in the beta frequency band
- Polygenic risk score analyses show that subjects with more beta power‐associated genetic variants have an increased risk of having GGE
- Mendelian randomization analyses suggest a causal effect of GGE genetic liability on beta oscillations.

## 1 INTRODUCTION

The power of oscillations in background electroencephalogram (EEG) is a highly stable and heritable human trait.^1^ It is easily acquired and can be automatically analyzed by software, rather than subjective interpretation. Epilepsy is highly heritable and is characterized by altered brain excitability.^2^, ^3^ Oscillatory activity is believed to serve an essential role in corticothalamic functioning, and can be measured as power of oscillations in background EEG at different broadband frequencies.^4^ Neurophysiological relationships between background EEG and generalized epileptiform discharges have been well described.^5^, ^6^, ^7^, ^8^ However, it is currently unknown whether background oscillatory activity is itself associated with epilepsy, and whether background EEG and epilepsy have a shared neurobiological and genetic basis.

There have been some studies where background EEG oscillation measurements have been directly compared between people with epilepsy and healthy controls. However, such studies have yielded conflicting results, most likely because sample sizes were small and antiseizure drugs can strongly affect EEG measurements.^9^, ^10^, ^11^, ^12^, ^13^, ^14^These limitations and bias can be overcome by large‐scale genetic studies, in which genetic determinants of background EEG measurements are assessed independently in healthy controls (presumably not taking antiseizure drugs). These genetic determinants can then be compared to genetic determinants of different epilepsy phenotypes, as assessed in a different study. Comparing these independent studies allows for a well‐powered and unbiased assessment of shared genetic determinants of epilepsy and EEG oscillations.

Here, we therefore assessed whether oscillatory background EEG is genetically correlated with focal and generalized epilepsy. The association between genetic variants and background brain activity was previously investigated in a genome‐wide association study (GWAS) on 8425 subjects without epilepsy.^15^ We combined these data with our recently published large GWAS of epilepsy,^16^ to examine genetic correlations between several types of epilepsy and oscillatory brain activity across frequency bands (delta, 1–3.75 Hz; theta, 4–7.75 Hz; alpha, 8‐12.75 Hz; and beta, 13–30 Hz). Next, we utilized polygenic risk scoring (PRS) to assess whether people with GGE have a genetic predisposition toward altered background brain activity. We then replicated genetic correlation and polygenic analyses using an independent cohort from the Epi25 Collaborative (*n* = 4851 people with epilepsy and 20 428 controls). Finally, we performed Mendelian randomization (MR) to gain insight into possible causal relationships between genetic variants associated with epilepsy and those associated with background EEG. We thus provide converging evidence for consistent cross‐trait genetic overlap between epilepsy and background EEG.

## 2 MATERIALS AND METHODS

### 2.1 Study population: Discovery dataset

The participants derived from the epilepsy GWAS^16^ for the current analyses were Caucasian subjects. The epilepsy GWAS included 13 control cohorts.^16^ Case/control ascertainment and diagnostic criteria were previously reported.^16^ As described previously,^16^ epilepsy specialists diagnosed people with epilepsy and ascertained phenotypic subtypes. Population‐based datasets, some of which had been screened to exclude neurological disorders, were used as controls. However, due to the relatively low prevalence of epilepsy in the general population (~0.5–1%), screening to exclude epilepsy in control cohorts will have only a minor effect on statistical power. Summary statistics from the recent epilepsy GWAS conducted by the International League Against Epilepsy (ILAE) Consortium on Complex Epilepsies GWAS were available for *n* = 12 803 cases (with either focal or generalized epilepsy) and 24 218 controls.^16^ From those participants, the following subjects were excluded for those analyses requiring individual‐level genotype data: Finnish ancestry (none had genetic generalized epilepsy [GGE]) and the subset of the EPICURE‐SP1 cohort that lacked informed consent for the current analyses, resulting in subject‐level genotype data being available for 11 446 people with epilepsy and 22 078 controls. Subjects with epilepsy were stratified into GGE (*n* = 3122) and focal epilepsy (*n* = 8324); GGE was further subdivided into childhood absence epilepsy (CAE; *n* = 561), juvenile absence epilepsy (JAE; *n* = 311), juvenile myoclonic epilepsy (JME; *n* = 1000), and generalized tonic–clonic seizures only (GTCS only; *n* = 195). GGE subtype information was not available for 1055 people with epilepsy.

We downloaded summary statistics of the ENIGMA‐EEG GWAS of resting state oscillation power in the delta (1–3.75 ​Hz), theta (4–7.75 Hz), alpha (8–12.75 Hz), and beta (13–30 Hz) bands at the vertex (Cz) electrode (*n* = 8425 participants).^15^ This EEG GWAS was based on five cohorts from four cooperating centers. Although the selection criteria varied across cohorts, all adult cohorts included epilepsy and prolonged unconsciousness after head trauma as exclusion criteria, which were communicated at the time of recruitment or at the first laboratory visit; because neurological disorders were an exclusion criterium, we do not expect subjects to be taking antiseizure drugs (although this was no explicit exclusion criterion). All these were self‐ or parent‐reported retrospective questions. A full sample description and recording specifics are available in the supplement of the original study,^15^ and the EEG analysis protocol is available online at http://enigma.ini.usc.edu/ongoing/enigma‐eeg‐working‐group/. In brief, eyes‐closed resting EEG was recorded or offline rereferenced to averaged earlobes, visually cleaned with standard criteria by local expert EEG analysts with rogue channels removed, and scanned for sleep transition (eye rolling, alpha dropout). Eye movement was removed using regression or independent component analysis. A minimum of 1 min of recording was required.

Approval for the source studies was obtained by all relevant institutional review boards, and all study participants provided written informed consent according to the Declaration of Helsinki.

### 2.2 Replication dataset

To replicate our findings, we used data from the Epi25 Collaborative (http://epi‐25.org/). This cohort currently comprises 4851 people with epilepsy, of whom 2612 have focal epilepsy and 2239 have GGE (no data on GGE subtypes were available). The cases were matched to a total of 20 428 controls from the Partners Healthcare Biobank (*n* = 14 857), the Epi25 Collaborative (*n* = 210), the Genetics and Personality consortium (*n* = 456), and an in‐house project on inflammatory bowel disease (*n* = 4905). The cohorts were genotyped on the Illumina Global Screening Array, with the exception of the Partners Healthcare Biobank participants, who were genotyped on the Illumina Multi‐Ethnic Screening Array. Approval was obtained by all relevant institutional review boards, and all study participants provided written informed consent according to the Declaration of Helsinki.

### 2.3 Genetic correlation analyses

Genetic correlations between epilepsy subtypes and oscillatory brain activity were computed using bivariate linkage disequilibrium score regression (LDSC).^17^ For these analyses, as no individual‐level genotype data were available from the EEG dataset, we used published summary statistics of the EEG frequency bands (alpha, beta, delta, and theta; *n* = 8425 participants) and the epilepsy subtypes (focal, GGE, CAE, JAE, JME, and GTCS only; *n* = 12 803 cases suffering from either focal or generalized epilepsy and 24 218 controls) from the ILAE consortium as a discovery dataset.^16^ For LDSC replication analyses, we used unpublished data from the Epi25 Collaborative (http://epi‐25.org/; *n* = 4851 people with epilepsy and 20 428 controls). For discovery and replication LDSC analyses, default settings of LDSC were used, with precomputed linkage disequilibrium (LD) score weights derived from the European subset of the 1000 Genomes project.^18^ See Table S1 for the number of single nucleotide polymorphisms (SNPs) per LDSC analysis. The significance threshold was Bonferroni‐corrected for the two main epilepsy subtypes studied (GGE and focal) but not for the EEG power spectra, because these were all highly correlated at *p *< 10^−17^ (Table S2), resulting in a significance threshold of *p *= .05/2 = .025. Similarly, we did not correct for the individual GGE subtypes, which are phenotypically similar and genetically highly correlated.^16^

### 2.4 PRS analyses

For PRS analyses, we used individual‐level genotype data derived from the epilepsy GWAS^16^ and summary statistics from the EEG GWAS.^15^ Quality control was performed as reported in the published epilepsy GWAS.^16^ We then added a genotype filter for call rate greater than .99 and the exclusion of genetically related subjects to allow for highly conservative PRS estimates. Genetic interrelatedness was calculated with KING,^19^ and one subject from each pair with third‐degree or higher relatedness (kinship coefficient > .0442) was excluded. PRSice^20^ was used with default settings to assess whether subjects with epilepsy had different EEG frequency power PRSs compared to controls. In brief, to each SNP we assigned a weight proportional to its association in the four EEG GWASs (alpha, beta, delta, and theta). Next, individual PRSs were calculated as the sum of weighted effect alleles for every subject from the epilepsy cohort. These PRSs were standardized with a *Z*‐score transformation : $\frac{\text{PRS}‐\text{mean}\left(\text{PRS}\right)}{\text{SD}\left(\text{PRS}\right)}$. SNPs were pruned to a subset of genetically uncorrelated SNPs (LD *R* ^2^ < .1), and PRS values were calculated using a number of different *p*‐value thresholds from .0001 to .5. Next, logistic regression analyses, corrected for sex and 10 genetic ancestry principal components (PCs), were performed to assess the association of these PRS scores with GGE. The PRS with the highest association with GGE was chosen as the "best fit," after which logistic regression analyses were repeated to assess the association of this PRS with the other epilepsy subtypes. We used a conservative *p *< .001 significance threshold to correct for multiple comparisons, as recommended for PRSice.^20^ Explained variance represented by the Nagelkerke *R* ^2^ was computed using a logistic regression of the PRS, subtracted from the baseline model (covariates only: sex and four PCs). To quantify the association of beta power PRS with GGE, we used PRSice standard settings to divide subjects into 10 deciles based on their beta power PRS scores. We then performed logistic regression to compare the risk of having GGE between every decile, with the lowest (0%–10%) as a reference (corrected for sex and four PCs). We then repeated the analyses in the independent Epi25 cohort. This dataset contained approximately one third fewer GGE cases than the discovery cohort, providing insufficient power to exactly replicate our discovery PRS findings. We therefore performed quasireplication using a one‐sample test of the proportion to assess concordance effect directions between discovery and replication PRS analyses, computing *Z*‐scores that were converted into *p*‐values:

$$Z=\frac{\hat{p}‐p_{0}}{\sqrt{p_{0}\left(1‐p_{0}\right)/n}}$$

where the *p* = the sample proportion; H_0_ represents the null hypothesis: *p* = *p*_0;_ and the alternative hypothesis H_1_ is p ≠ p_0_.

### 2.5 Mendelian randomization

Two major limitations of observational studies and other types of studies are unmeasured confounding and uncertainties about cause and effect. MR has the potential to overcome these limitations, as MR leverages genetic instruments (most often SNPs) as exposures as well as outcomes. Because SNPs are not influenced by state‐dependent factors, MR has the potential to shed light on potential causal mechanisms between two traits; SNPs strongly associated with two or more traits index these traits without confounding. MR can be done in two directions for two given traits, with each MR analysis testing whether one trait has a potential effect on the other. However, here, we could only conduct one‐way MR due to lack of genome‐wide significant loci in the EEG GWAS. Several MR techniques are available, and the consensus is that results from different approaches show robustness and consistency of results across methods.

To explore possible causal effects of GGE genome‐wide loci (exposure) on EEG background oscillations (outcome), we thus conducted MR analyses using GGE and EEG summary statistics data. Two hundred twenty‐eight SNPs significantly associated with GGE (*p* < 5 × 10^−8^) were extracted from both the GGE and EEG GWASs. The summary statistics of 228 SNPs were harmonized to ensure the SNP effect direction corresponded with equal effect alleles across GGE and EEG. We used the “TwosampleMR” package^21^ in R to perform fixed effects inverse variance‐weighted (IVW), weighted median, and MR Egger models. We then performed sensitivity analyses, including horizontal pleiotropic effects estimated by the intercept of MR Egger, residual heterogeneity due to pleiotropy estimated by Cochran Q test,^22^ and leave‐one‐out analyses (for the fixed effects IVW model), to evaluate whether any single instrumental variable was driving the results. Generalized summary data‐based MR (GSMR) analyses were performed using the “GSMR”^23^ package in R. To that end, first the LD matrix of the selected SNPs was calculated using PLINK^24^ and GCTA^25^ within 1000 Genomes Phase 3 data.^18^ The minimum number of instrumental variables in the GSMR model was loosened from 10 to five as there were only eight independent (*r* ^2 ^< .01, LD window = 10 Mb) significant loci identified in the GWAS of GGE (and none in the EEG GWAS). We used default options in GSMR with heterogeneity in dependent instruments (HEIDI) testing for instrumental outliers’ detection. At the end, we repeated GSMR with loosened LD prune thresholds (i.e., *r* ^2^ < .1, *r* ^2^ < .15, and *r* ^2^ < .2), because GSMR takes LD structure into account by adding the LD matrix. The significance threshold was Bonferroni corrected for all seven of these MR models (*p *= .05/7 = .007).

### 2.6 Data availability

GWAS summary statistics used for the current analyses are available online: http://enigma.ini.usc.edu/research/download‐enigma‐gwas‐results/; http://www.epigad.org/gwas_ilae2018_16loci.html.

## 3 RESULTS

### 3.1 Genetic correlations between epilepsy and oscillatory brain activity

**FIGURE 1:**
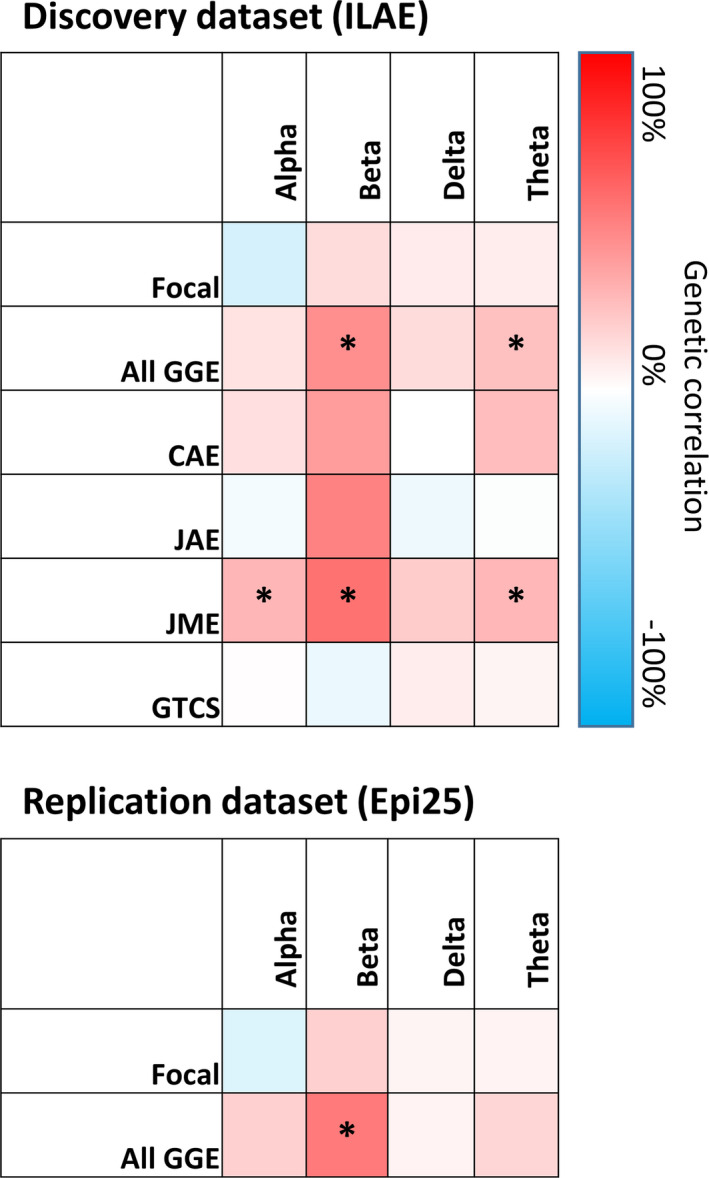
Genetic correlations between electroencephalographic (EEG) frequency bands and epilepsy subtypes. Genetic correlations were calculated by comparing the EEG frequency band genome‐wide association study (GWAS) with the International League Against Epilepsy (ILAE) GWAS (upper panel, discovery dataset) and the Epi25 GWAS (lower panel, replication dataset). * *p *< .05. CAE, childhood absence epilepsy; GGE, genetic generalized epilepsy; GTCS, generalized tonic–clonic seizures; JAE, juvenile absence epilepsy; JME, juvenile myoclonic epilepsy

In a total study population of 45 446 subjects (*n* = 8425 from the EEG and *n* = 37 021 from the epilepsy GWASs), we computed genetic correlations (Rg) of alpha, beta, delta, and theta oscillatory brain activity with focal epilepsy and GGE. We found significant correlations between GGE and beta power (Rg = 0.44 ± SE of .18, *p *= .01) and theta power (Rg = 0.25 ± 0.11, *p *= .02; Figure 1, upper panel, Table S1). This was further supported by the correlations between beta power and theta power with the GGE subtypes CAE, JAE, and JME; all had similarly high correlation coefficients. We found no genetic correlations between focal epilepsy and any of the EEG phenotypes. We then attempted to replicate the genetic correlations using the unpublished Epi25 dataset and found genetic correlations similar (in both sign and effect size) to the discovery analyses (Figure 1, lower panel); GGE correlated with beta power (Rg = 0.52 ± 0.21, *p *= .01), whereas the genetic correlation between theta power and GGE paralleled the discovery cohort (albeit not reaching significance: Rg = 0.16 ± 0.12, *p *= .18). All genetic correlation estimates with focal epilepsy were again nonsignificant. There were no data available for GGE subtypes.

### 3.2 Oscillatory brain activity polygenic scores are associated with generalized epilepsy

**FIGURE 2:**
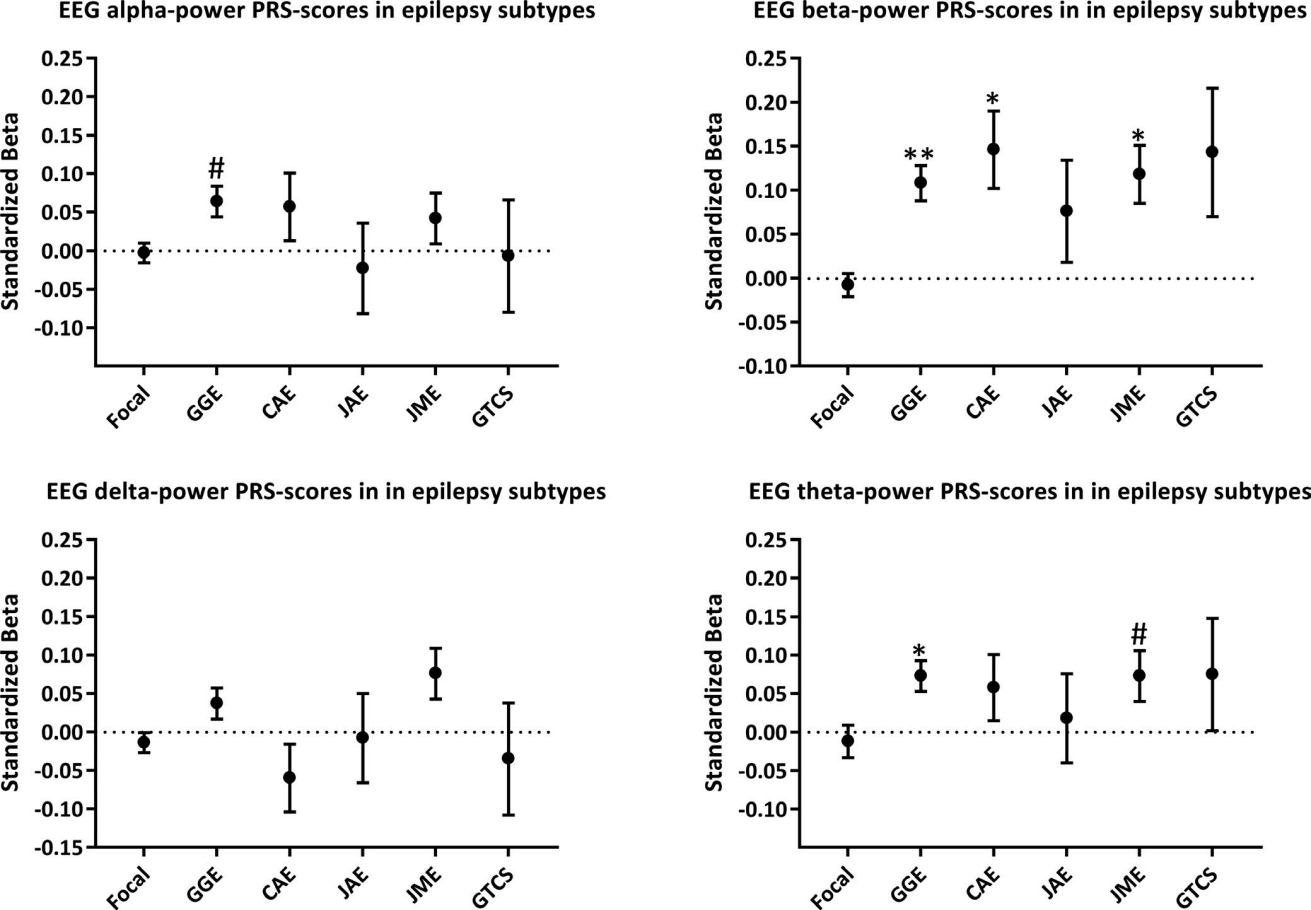
(A) Beta and theta power electroencephalographic (EEG) oscillation polygenic risk scores (PRSs) are associated with generalized epilepsy but not with focal epilepsy. The "best‐fit" *p*‐value threshold (*p*_t_) was chosen based on the most significant association with genetic generalized epilepsy (GGE), which was then applied to all other epilepsy subtypes. The numbers of single nucleotide polymorphisms included in each model were 2670 for alpha power (*p*_t_ = .0105), 10 861 for beta power (*p*_t_ = .06245), 8182 for delta power (*p*_t_ = .0446), and 3833 for theta power (*p*_t_ = .01665). Logistic regression analyses were performed to assess the association between the PRSs and the different epilepsy subtypes, corrected for sex and 10 principal components. #*p *< .05, *p < .001, **p < 10^−7^. Childhood absence epilepsy (CAE), generalized tonic–clonic seizures only (GTCS), juvenile absence epilepsy (JAE), and juvenile myoclonic epilepsy (JME) are GGE subtypes. Focal, focal epilepsy

**FIGURE 3:**
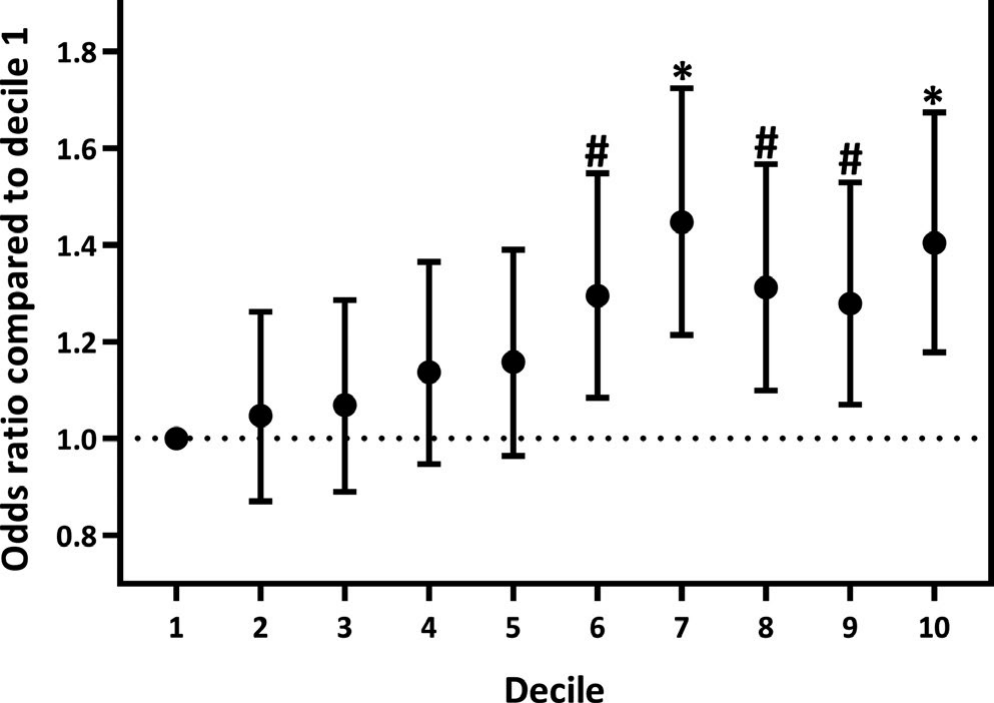
Polygenic risk score (PRS) analyses show that higher beta‐power PRS is associated with an increased likelihood of having GGE. All subjects were divided into 10 deciles based on their beta‐power PRS scores. Logistic regression analyses were performed to quantify the increased risk of having GGE between every decile compared to the lowest decile (0–10%) as a reference. The odds ratios of these analyses are displayed on the Y‐axis. #*p*<.05; *p<.001

We used polygenic scoring to utilize the full distribution of background EEG‐associated SNPs to assess whether people with epilepsy have a different polygenic score for specific frequency bands compared to controls. We observed significant positive associations between beta and theta power PRSs with GGE, in line with the LDSC results (Figure 2). In particular, beta power PRSs were strongly associated with GGE (beta = .11, SE = .020, *p *= 5.3 × 10^−8^, explained variance = .21%; Figure 2), which was further supported by significant associations of beta power PRS with its subtypes CAE (beta = .15, SE = .044, *p* = 8.5 × 10^−4^) and JME (beta = .12, SE = .033, *p *= 3.6 × 10^−4^). Furthermore, of the participants in the GGE case–control cohort, those in the highest 10% decile of beta power PRS scores were 1.4‐fold more likely to have GGE compared to the people in the lowest 10% PRS decile (Figure 3; odds ratio [OR] = 1.40, 95% confidence interval [CI] = 1.18–1.67, *p* = 1.5 × 10^−4^). When using the independent Epi25 cohort as a replication dataset, we found that the directions of effect agreed with the discovery analyses for all associations between EEG PRSs and GGE (*p*_one‐sided_ = .023, *p*_two‐sided_ = .046; Figure S1). EEG PRSs were not significantly different between people with focal epilepsy and controls.

### 3.3 MR analyses

MR analyses were performed to assess potential causative relationships between background EEG and GGE. Eight GGE‐associated SNPs were selected as instrumental variables at a strict LD prune threshold (*r* ^2^ < .01, LD window = 10 Mb). These were used in fixed effects IVW, weighted median, MR Egger, and GSMR (*r* ^2^ < .01) models. After loosening the LD threshold, 11 (*r* ^2^ < .1), 12 (*r* ^2^ < .15), and 14 (*r* ^2^ < .2) SNPs were selected as instrumental variables for GSMR models. Causal effects of GGE loci on beta oscillations were found at the LD *r* ^2^ < .15 and *r* ^2^ < .2 thresholds (OR = 1.79, 95% CI = 1.189–2.707, *p* = 5.2 × 10^−3^ and OR = 1.723, 95% CI = 1.180–2.516, *p* = 4.8 × 10^−3^, respectively; Table S4, Figure S2). Significant heterogeneity was detected in the fixed effects IVW model (Q‐statistic = 18.188, *df* = 7, *p *= .01) and MR Egger model (Q‐statistic = 14.594, *df* = 6, *p *= .02). No SNPs altered the pooled β coefficient in the leave‐one‐out sensitivity analysis (β = .374, *p *= .314) in the fixed effects IVW model. We found no evidence of horizontal pleiotropic effects. Similarly, the HEIDI test detected no SNPs as pleiotropic outliers.

## 4 DISCUSSION

Here, we leveraged the largest currently available GWASs to assess shared genetic underpinnings of epilepsy and of background EEG oscillations. In particular, we found strong genetic relationships between GGE and beta power oscillations, which were replicated in an independent sample.

Previous studies comparing EEG background oscillations between people with epilepsy and controls are inconsistent; some show increased power in all frequency bands (alpha, beta, delta, theta), whereas others show only increases in specific frequency bands or even decreases in power.^9^, ^10^, ^11^, ^12^, ^13^, ^14^ This heterogeneity likely reflects multiple variables that are difficult to control for in clinical studies, such as antiepileptic drug (AED) usage, sleep deprivation, influence of (inter‐)ictal epileptic brain activity, EEG processing, and electrode placement. We overcame such limitations by determining the genetic underpinnings of EEG frequency bands in people without epilepsy who are AED‐naive, and with consistent electrode placement and signal processing. We applied several statistical models to assess this overlap and found that people with generalized, but not focal, epilepsy carry a relative abundance of genetic variation associated with higher beta oscillations. MR analyses pointed to causal effects of genetic liability to GGE on beta power.

We did not find genetic correlations between background EEG and focal epilepsy. Although power was limited for this analysis, this finding is consistent with the low contribution of common genetic variants in focal epilepsy and the lack of genetic overlap between focal and generalized epilepsy.^16^ Focal epilepsy is likely to represent a more heterogenous group of different causes of epilepsy, many of which do not have a primary genetic cause (e.g., symptomatic epilepsy after traumatic brain injury). Moreover, focal epilepsy by definition only affects one part of the brain and is therefore less likely to be associated with germline genetic variation and background EEG oscillations, which most likely affect the whole brain. Although we found associations of common variants with focal epilepsy in our latest GWAS, the overall polygenic burden and SNP‐based heritability was modest compared to GGE.^16^ This suggests that further studies assessing common genetic variants in focal epilepsy are less likely to yield major advances. Perhaps further studies on smaller, more homogenous focal epilepsy cohorts or studies assessing rare genetic variants could yield more insights into its pathophysiology. In contrast to focal epilepsy, the EEG discharges that characterize generalized epilepsy are dependent on the thalomocortical system.^5^, ^26^ Similarly, background oscillations have been functionally attributed to the thalamocortical system,^27^, ^28^ suggesting that thalamocortical functioning could represent a common neurobiological mechanism reflecting overall brain excitability, which influences both GGE risk and (beta power) background oscillations.

Our results should be interpreted in the light of several limitations. First, we are aware of the possible advantages of using genome complex trait analysis (GCTA) relative to LDSC, but because no subject‐level genotype data are available for the EEG GWAS, we restricted our genetic correlation estimates to LDSC, which is based on summary statistics. LDSC has proven to be a reliable method for genetic correlation estimates, and results between LDSC and GCTA have proven consistent. Second, we found that the same genetic variants underlie both GGE and beta power oscillations, but our study does not prove that people with GGE have altered background oscillations, because we did not have EEG measurements of people with epilepsy in this study. Third, only one‐way MR analyses were performed due to lack of genome‐wide significant loci in the EEG GWAS. Our results suggest that GGE causally influences beta power oscillations. However, we cannot exclude the possibility of bidirectional causality between EEG and GGE, and thus it could also be possible that beta power has a causal effect on GGE risk. Fourth, we had insufficient data available to carry out subgroup analyses on ​subjects with nonlesional focal epilepsy.

Altogether, our results point to shared biological mechanisms underlying background EEG oscillations and the susceptibility for generalized seizures. Our findings thus open avenues to investigate the clinical utility of background oscillations in genetic generalized epilepsy. Potentially, prospective studies could confirm whether altered beta oscillatons could be a prodromal state of GGE or whether aberrant beta oscillations constitute a feature of epilepsy. Future studies may also integrate transcranial magnetic stimulation–EEG and/or event‐related potentials to examine whether beta and theta powers correlate with altered brain excitability in subjects with high epilepsy liability. We hypothesize that the genetic correlation between GGE and background oscillations will be reflected by measurable differences in background EEG measures between people with and without GGE, which could be used in the diagnostic workup after a first suspected seizure. This information can be used in machine‐learning studies by integrating background EEG with other sources of clinical and demographic data, which may one day increase the accuracy of epilepsy diagnosis.

## CONFLICT OF INTEREST

None of the authors has any conflict of interest to disclose.

## AUTHOR CONTRIBUTIONS

R.S., J.J.L., D.Sm., and B.P.C.K. contributed to the conception and design of the study. R.S., J.J.L., B.D.L., D.Sm., and B.P.C.K. contributed to the acquisition and analysis of data. R.S., J.J.L., B.D.L., C.L., D.L., A.S., D.Sc., J.A.C., K.R., R.A.R.B., O.D., K.P.J.B., F.E.J., D.Sm., and B.P.C..K. contributed to the drafting of the manuscript and preparing the figures. Members of the ILAE Consortium on Complex Epilepsies and EPI25 Collaborative contributed clinical and genetic data.

## Supporting information

Supplementary Material
